# Supplementary figures and images for: A multiomic approach to defining the essential genome of the globally important pathogen Corynebacterium diphtheriae
Source: PLoS Genet. 2023 Apr 26;19(4):e1010737. doi: 10.1371/journal.pgen.1010737 (PMC10166564; doi:10.1371/journal.pgen.1010737)

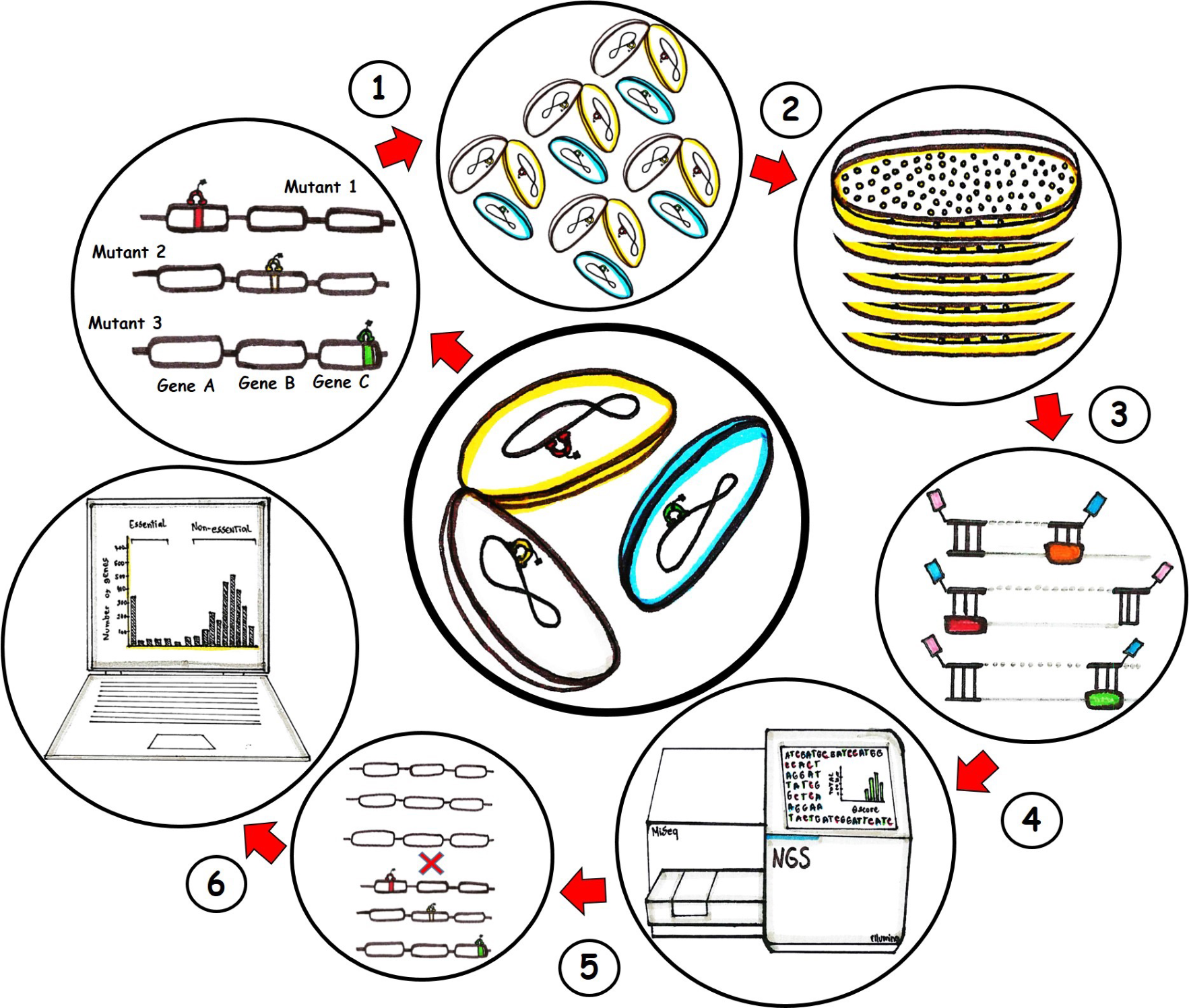

Supplement: S1 Fig — Construction of a high-density transposon insertion library of 0,6 million single mutations (1), cultivation under a best condition for a bacterial library growth (2) followed by DNA extraction and sample preparation with adaptors ligation at the transposon junctions (3) for the simultaneous sequencing with a next generation sequencer (Illumina platform) (4). The sequence data is compared with the wild type genome (5) in order to identify the essential and non-essential genes, revealing the fitness contribution of each gene under the condition analyzed (6). (TIF) [file pgen.1010737.s013.tif]

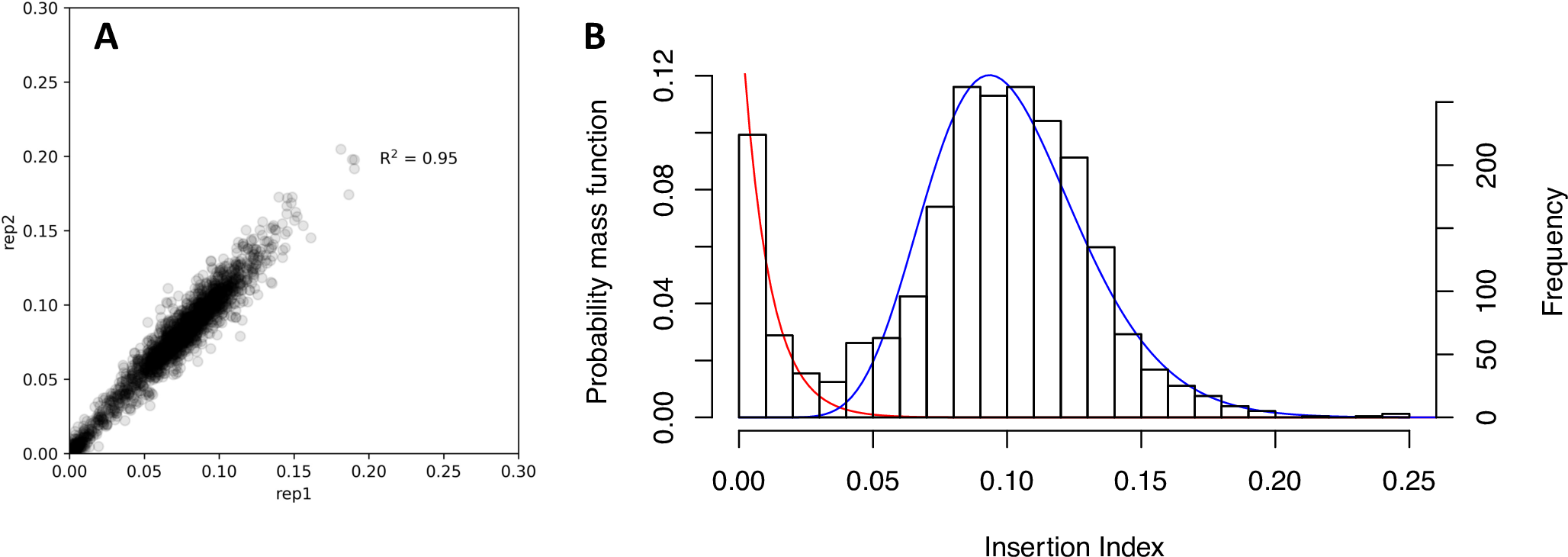

Supplement: S2 Fig — (A) Comparison of the insertion index scores of two technical replicates of the transposon mutant library. (B) Bi-modal distribution of the total insertion index scores for the transposon library. The exponential distribution fit to the left mode includes the essential genes (red), and the gamma distribution fit to the right mode captures the nonessential genes (blue). (TIF) [file pgen.1010737.s014.tif]

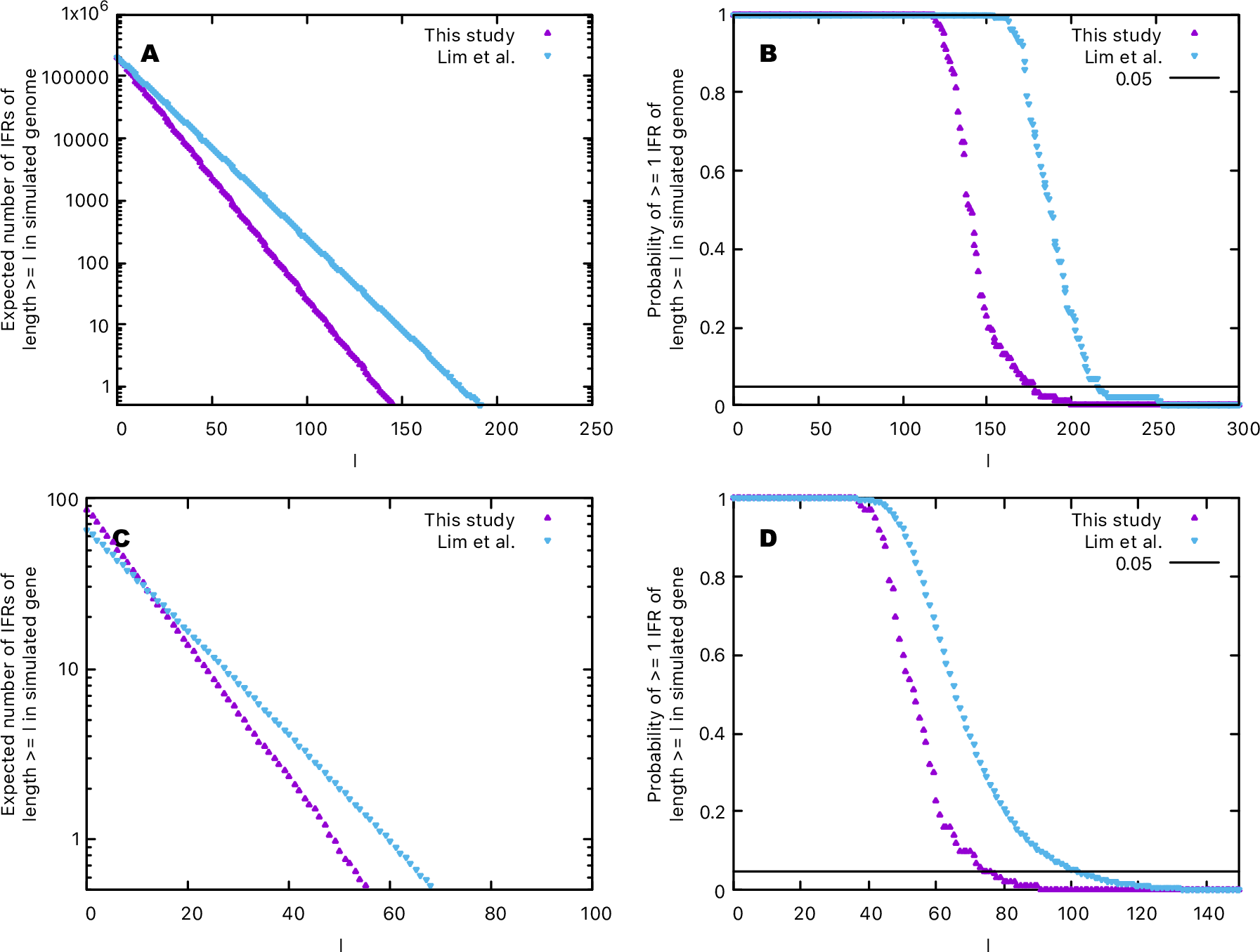

Supplement: S3 Fig — (A) The expected number of insertion-free regions (IFRs) of length l in a genome of a given size (here, 2,404,936 bp purple, and 3,079,253 bp blue, corresponding with the C. diphtheriae and C. glutamicum reference genomes used respectively) under the null model of N random insertions. The number of unique insertions was used as parameter ‘N’, plot A depicts the expected outcome of the null model over 100 simulations. (B) The related probability of at least one IFR of length l occuring anywhere in the simulated genome. (C and D). The same calculations repeated but within a simulated string of DNA of length g = 1,000, (equating to an IFR within a gene of 1,000 bp), repeated over 105 simulations. (TIF) [file pgen.1010737.s015.tif]

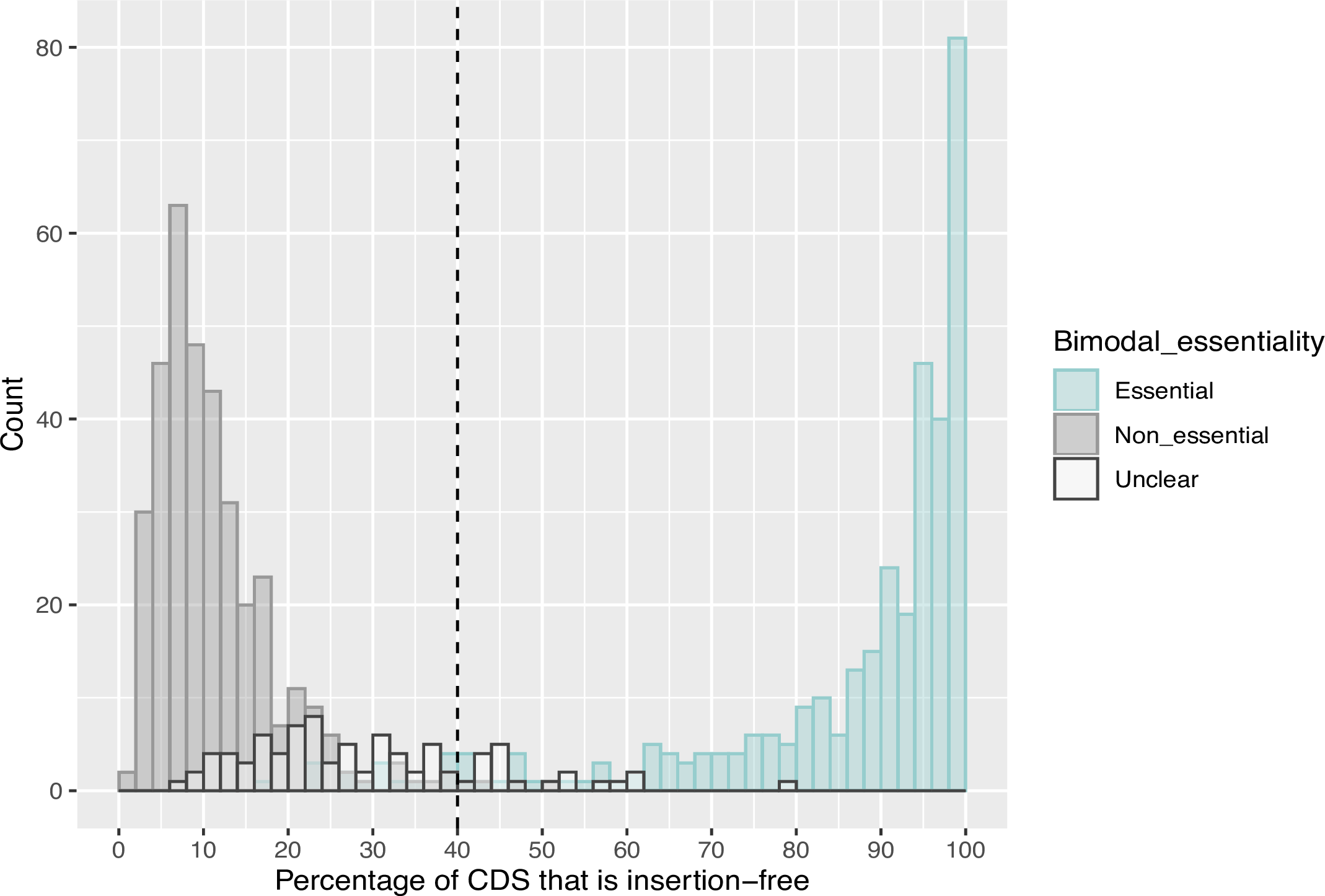

Supplement: S4 Fig — Statistically significant insertion-free regions (IFR; > = 74 bp, pgene = 0.05) within annotated genes were calculated as a percentage of the coding sequence (CDS). Genes without a statistically significant IFR were discarded from our analysis. Where a gene had more than 1 significant IFR, the length of the IFRs were summed. The percentage of each CDS that is significantly undisrupted was calculated for each gene and coloured according to the essential classification derived from the bi-modal analysis. We applied a threshold of 40% (dashed line). Genes with >40% of the CDS undisrupted by transposon mutagenesis, but not previously identified as essential (green) were identified as domain-essential genes. (TIF) [file pgen.1010737.s016.tif]

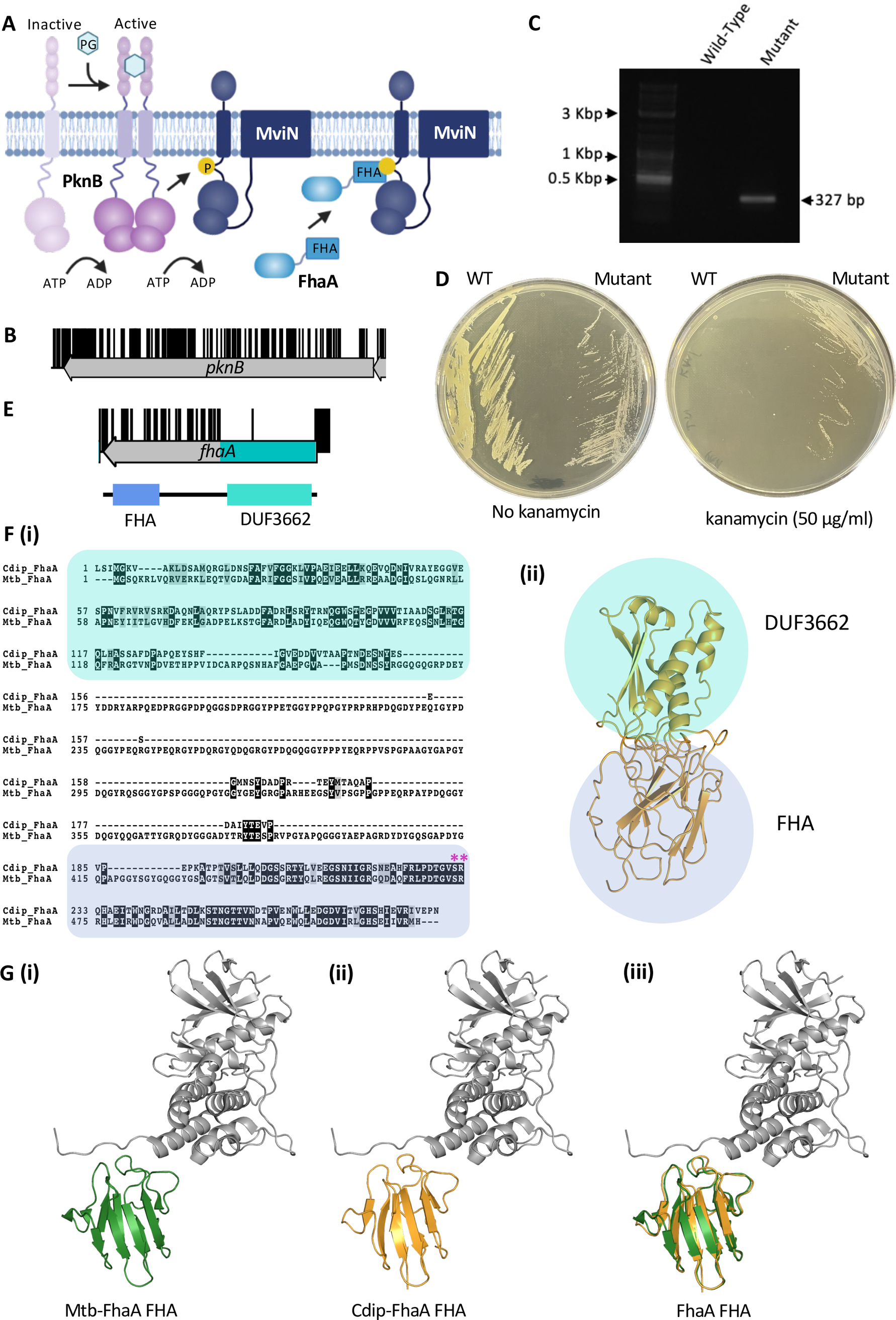

Supplement: S5 Fig — (A) Model of PknB phosphorylation of MviN pathway in M. tuberculosis adapted from Gee et al. (2012). (B) Transposon insertion profile of the phosphokinase pknB in C. diphtheriae. (C) PCR analysis of Wild-Type C. diphtheriae ISS3319 and C. diphtheriae ISS3319::pK18mobDIP0053 genomic DNA showing the presence of the 327 bp amplicon that indicates insertion of pK18mobDIP0053 in to the chromosome of ISS3319, which is absent from the WT strain (Markers: Lane 1: NEB 1Kb plus DNA ladder). (D) Wild-Type (WT) Corynebacterium diphtheriae and ISS3319::pK18mobDIP0053 (“Mutant”) on BHI medium with no kanamycin (left) and kanamycin selection (50 μg/ml) (right) on BHI medium indicating stable insertion of pK18mobDIP0053 in to the chromosome. (E)Transposon insertion data of fhaA capped at a frequency of 1. Protein domains are drawn beneath the gene track. (F) (i) Sequence comparison between Cdip-FhaA and Mtb-FhaA with both the domain of unknown function, DUF3662, and Fork-head associated (FHA) domain highlighted in coloured boxes. The conserved Ser473 and Arg474 of Mtb-FhaA that interact with P-Thr97 of MviN are highlighted with an asterisk (*). (ii) A prediction of the Cdip-FhaA structure with the DUF3662 and FHA domains highlighted. (G) (i) The solved structure of the FHA domain of Mtb-FhaA (green) interacting with the cytoplasmic domain of Mtb-MviN (grey; PDB: 3OUN). (ii) A model of Cdip-FhaA FHA (amber) alongside Mtb-MviN, and (iii) an overlay of both Cdip- and Mtb-FHA domains for structural comparison. (TIF) [file pgen.1010737.s017.tif]

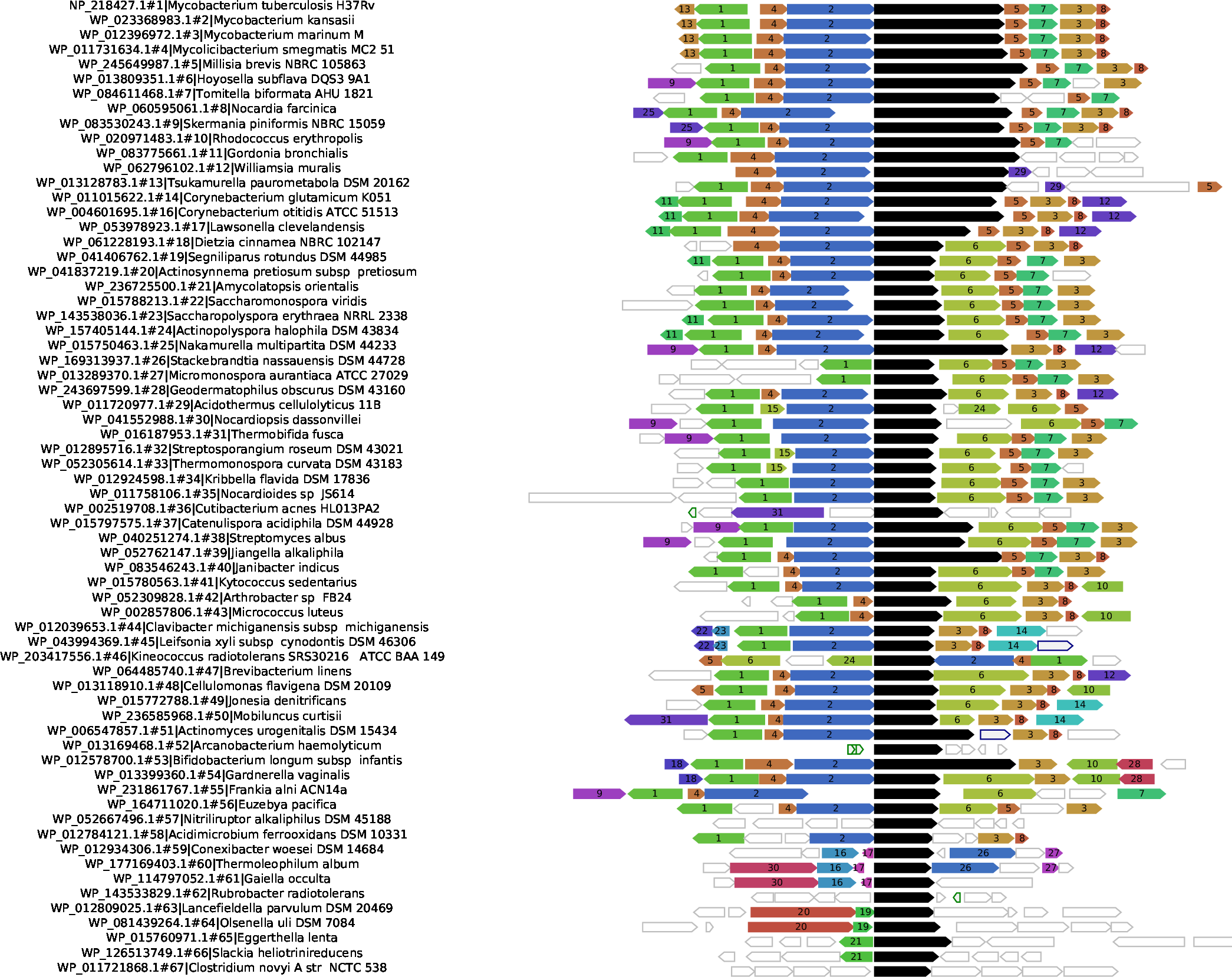

Supplement: S6 Fig — Gene neighbourhood of diphtheriae_02308 (mviN) orthologs (black) in representative Actinobacteria species, figure generated using FlaGs. Gene (6) is annotated as a “protein kinase family protein” and is frequently observed downstream from mviN orthologs that do not have an extended sequence. The remaining predicted gene functions associated with each number are provided in Supplementary Information. (TIF) [file pgen.1010737.s018.tif]

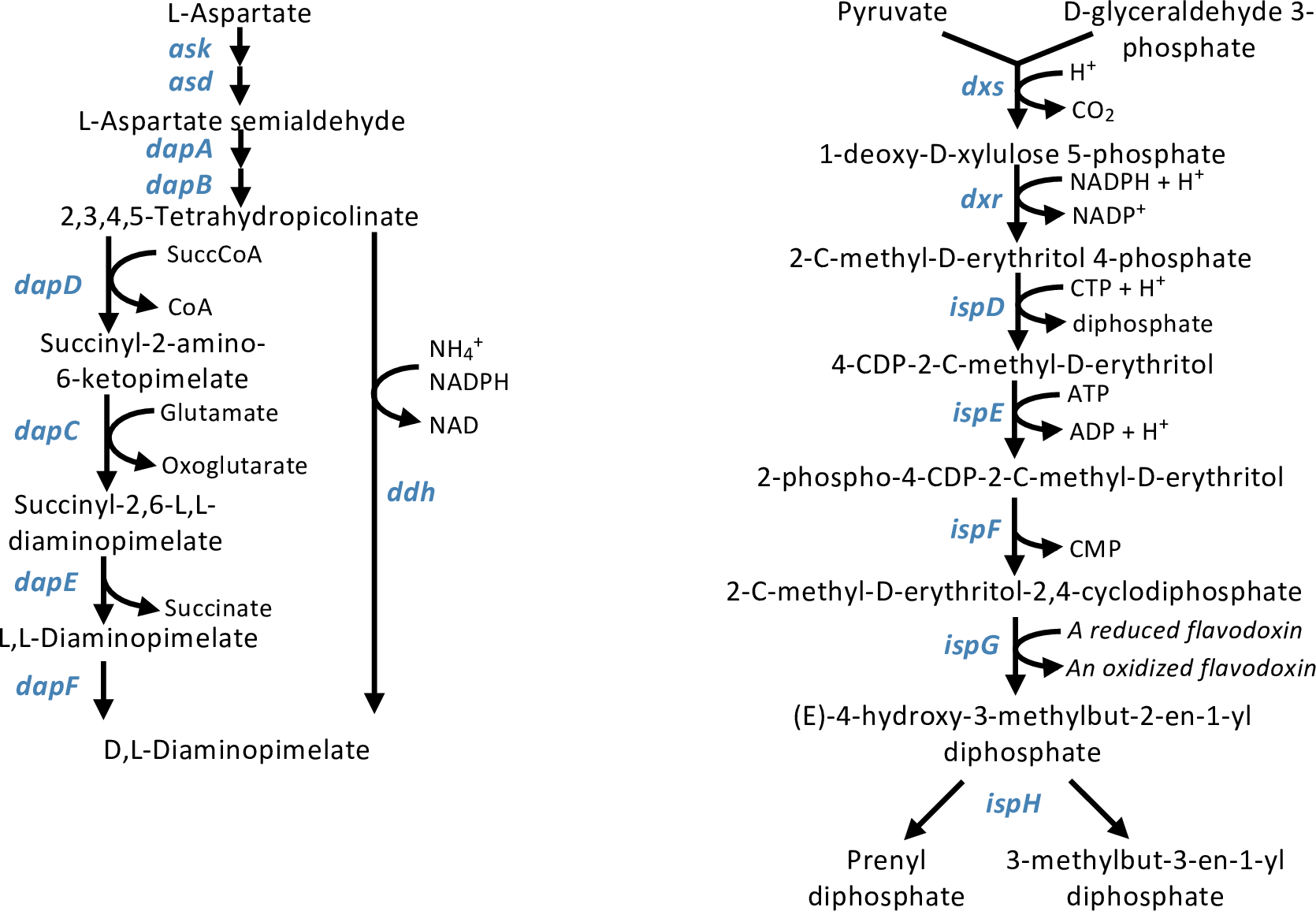

Supplement: S9 Fig — The diaminopimelate biosynthesis pathway and the methyl erythritol phosphate pathway of isoprenoid synthesis. The genes encoding the enzymes required for each step are coloured in blue. (TIF) [file pgen.1010737.s021.tif]

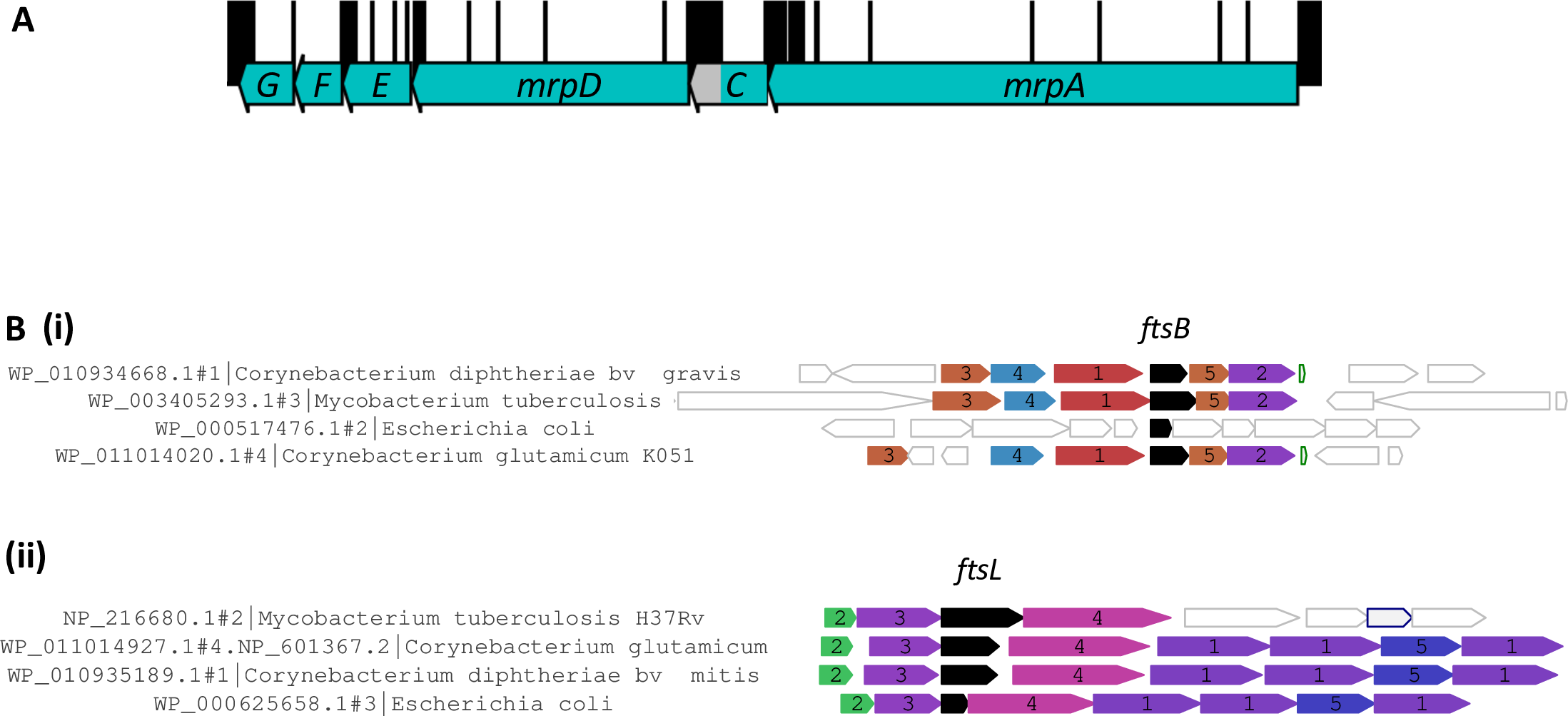

Supplement: S10 Fig — (A) Transposon insertion frequency within the mrp operon, with essential genes and regions highlighted in teal. (B) (i) The gene neighbourhood of ftsB (ftsL_1; diphtheriae_00817) homologs (black), figure generated by webFlaGs. The numbered genes encode: 1. phosphopyruvate hydratase; 2. Ppx/GppA family phosphatase; 3. nucleotide pyrophosphohydrolase; 4. lytic murein transglycosylase; 5. DUF501 domain-containing protein. (ii) The gene neighbourhood of ftsL (ftsL_2; diphtheriae_01542) homologs (black), figure generated by webFlaGs. The numbered genes encode: 1. UDP-N-acetylmuramoyl-L-alanine—D-glutamate ligase; 2. division/cell wall cluster transcriptional repressor MraZ; 3. rRNA small subunit methyltransferase H RsmH; 4. penicillin-binding membrane protein; 5 phospho-N-acetylmuramoyl-pentapeptide-transferase. (TIF) [file pgen.1010737.s022.tif]

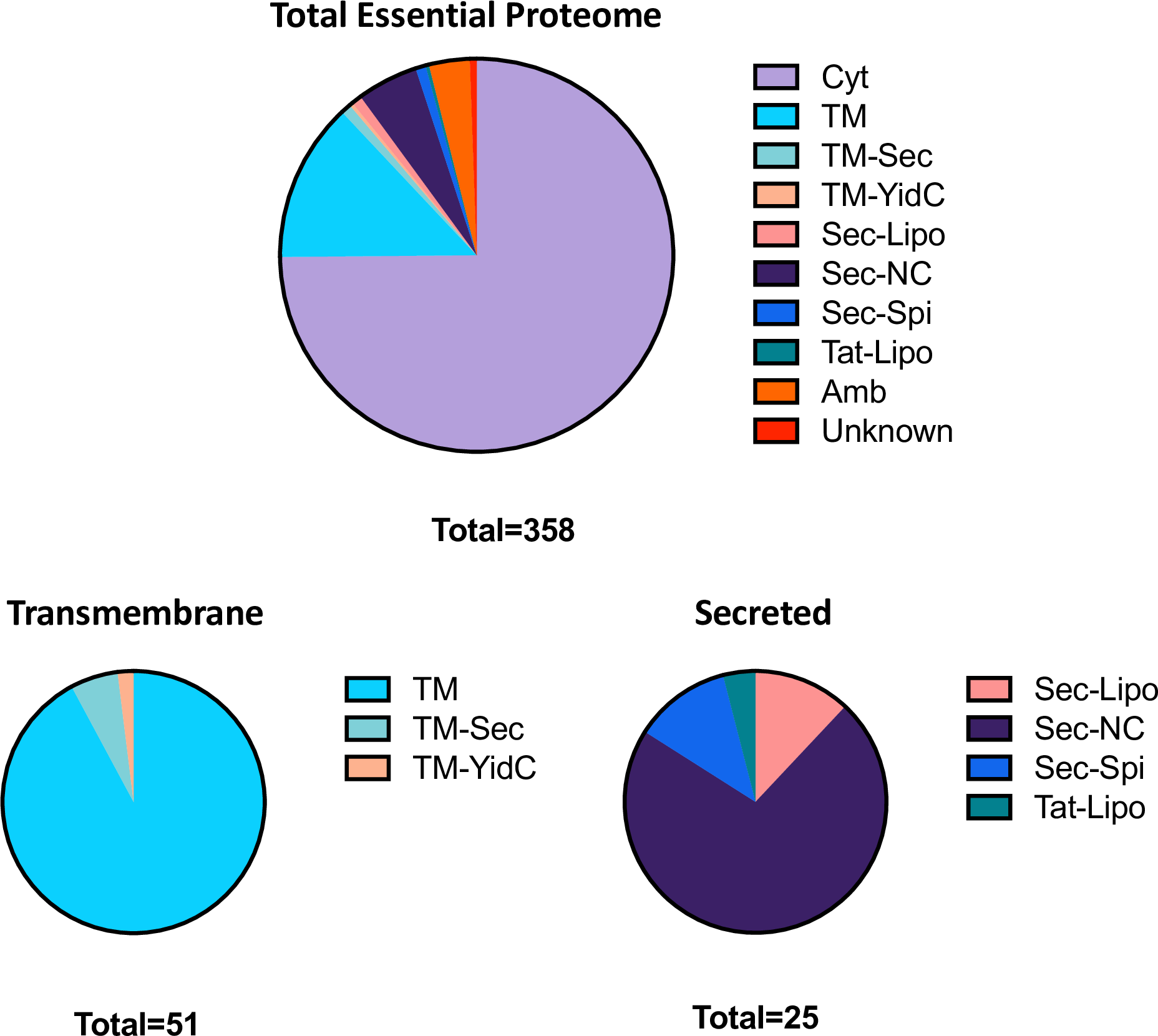

Supplement: S11 Fig — The predicted localization of essential proteins adapted from Sangal et al. (2015) shown for the total proteome (top), the predicted transmembrane proteins (left, 51) and predicted secreted proteins (right, 25). Abbreviations: Cyt, cytoplasmic; TM, transmembrane; Sec, secreted; Lipo, lipoprotein; NC, non-classical secreted protein; Spi, SpI type signal peptide; Tat, Tat signal peptide; Amb, ambiguous. (TIF) [file pgen.1010737.s023.tif]

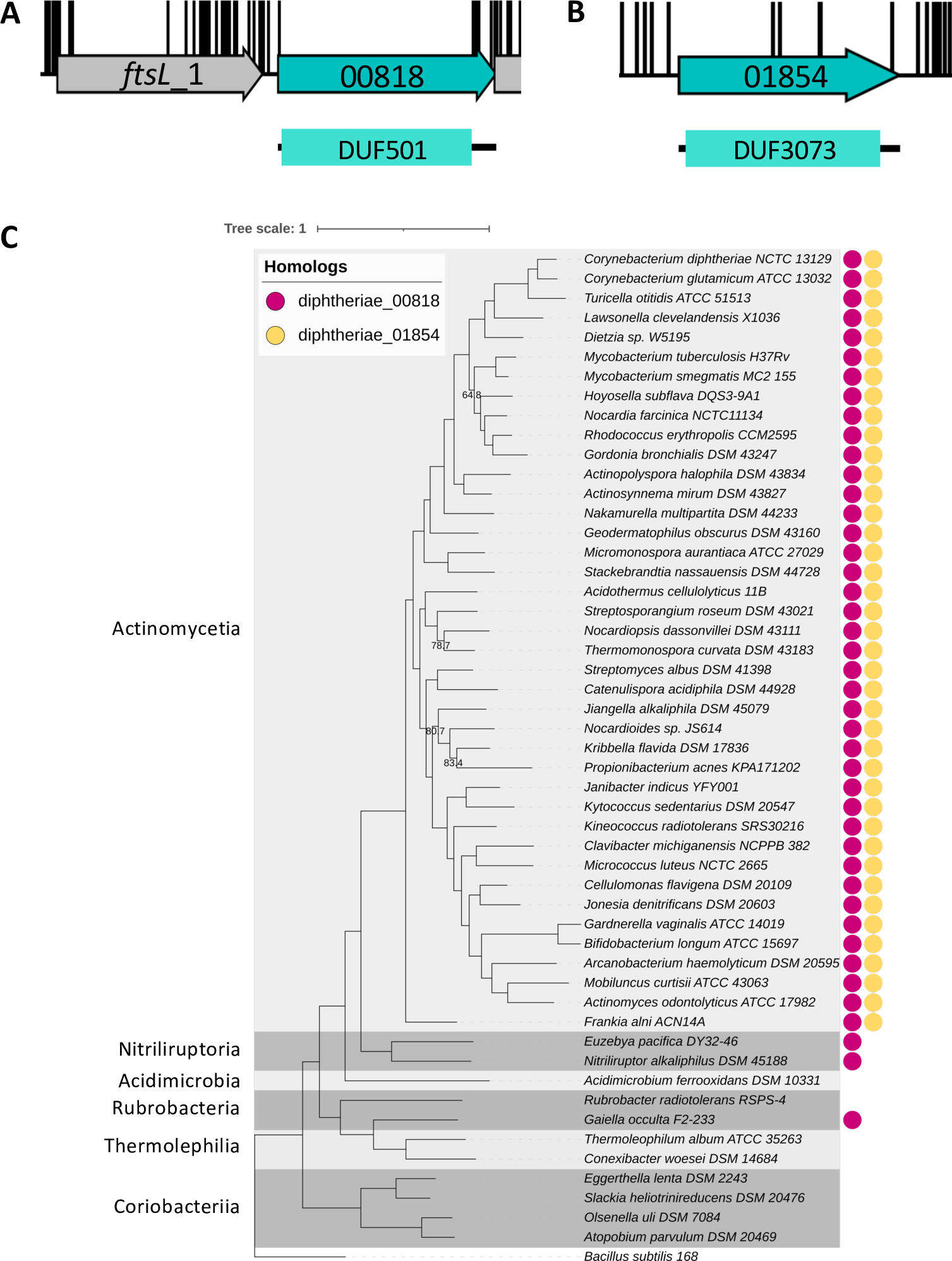

Supplement: S12 Fig — (A) and (B) Transposon insertion data for diphtheriae_00818 and diphtheriae_01854 genes with the respective domains of unknown function (DUF) DUF501 (PF04417) and DUF3073 (PF11273) displayed beneath. Transposon insertion sites are represented by vertical black bars, capped at a frequency of 1. (C) Distribution of homologs of diphtheriae_00818 and diphtheriae_01854 within representative genomes of the Actinobacteria phylum. Bacillus subtilis was used as an outgroup for construction of the tree; only bootstrap values under 100 are shown on the tree. Species are shaded by Class. The presence of a homolog, identified by BLASTP, is indicated by a coloured circle. (TIF) [file pgen.1010737.s024.tif]

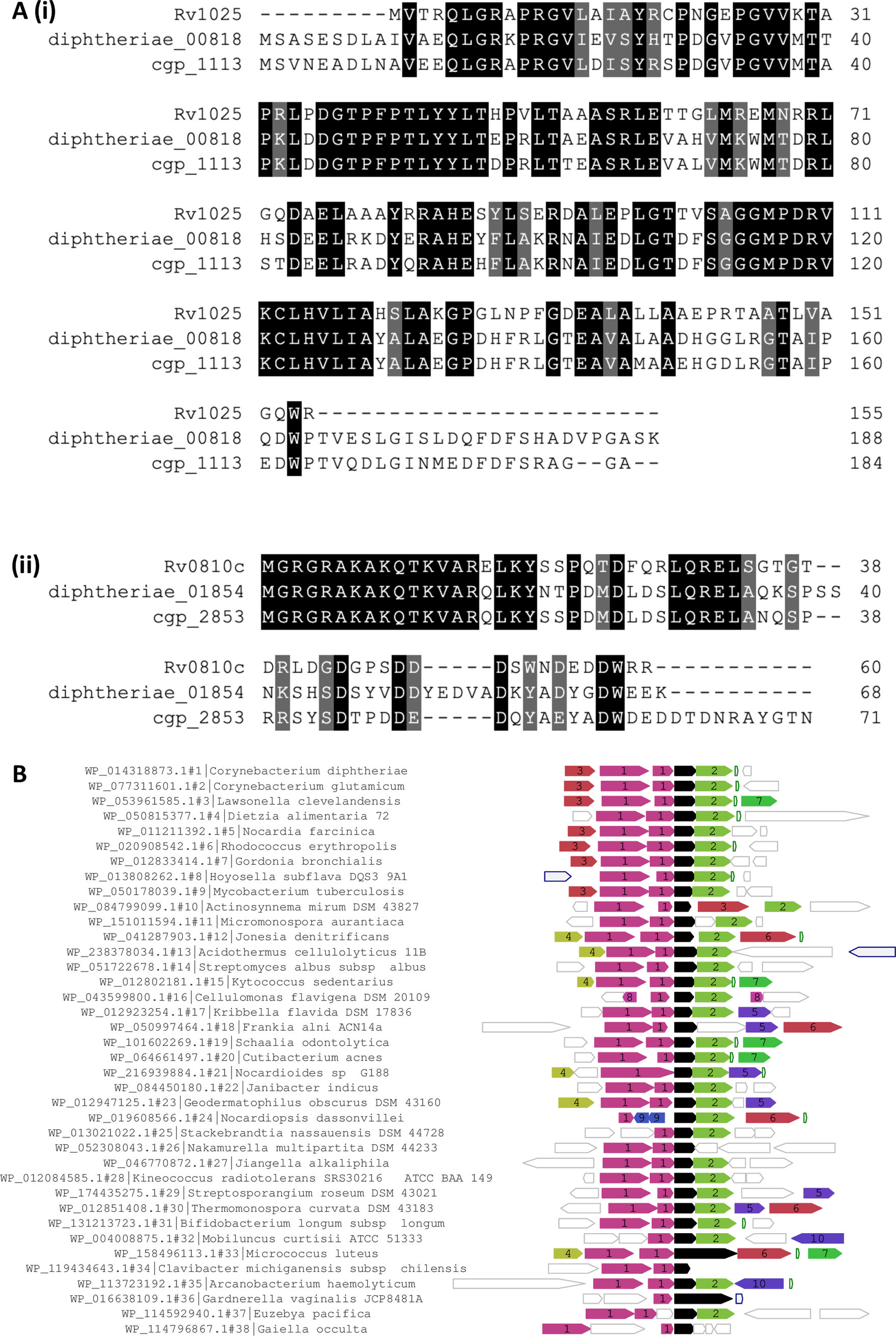

Supplement: S13 Fig — (A) Protein sequence alignment of the essential homologs of (i) diphtheriae_00818 and (ii) diphtheriae_01854 conserved in M. tuberculosis and C. glutamicum. Alignments generated using EMBL-EBI MUSCLE, conserved residues are shaded. (B) Gene neighbourhood of diphtheriae_00818 homologs (shown in black) in representative Actinobacteria species, figure generated using FlaGs. The remaining predicted gene functions associated with each number are provided in Supplementary Information. (TIF) [file pgen.1010737.s025.tif]

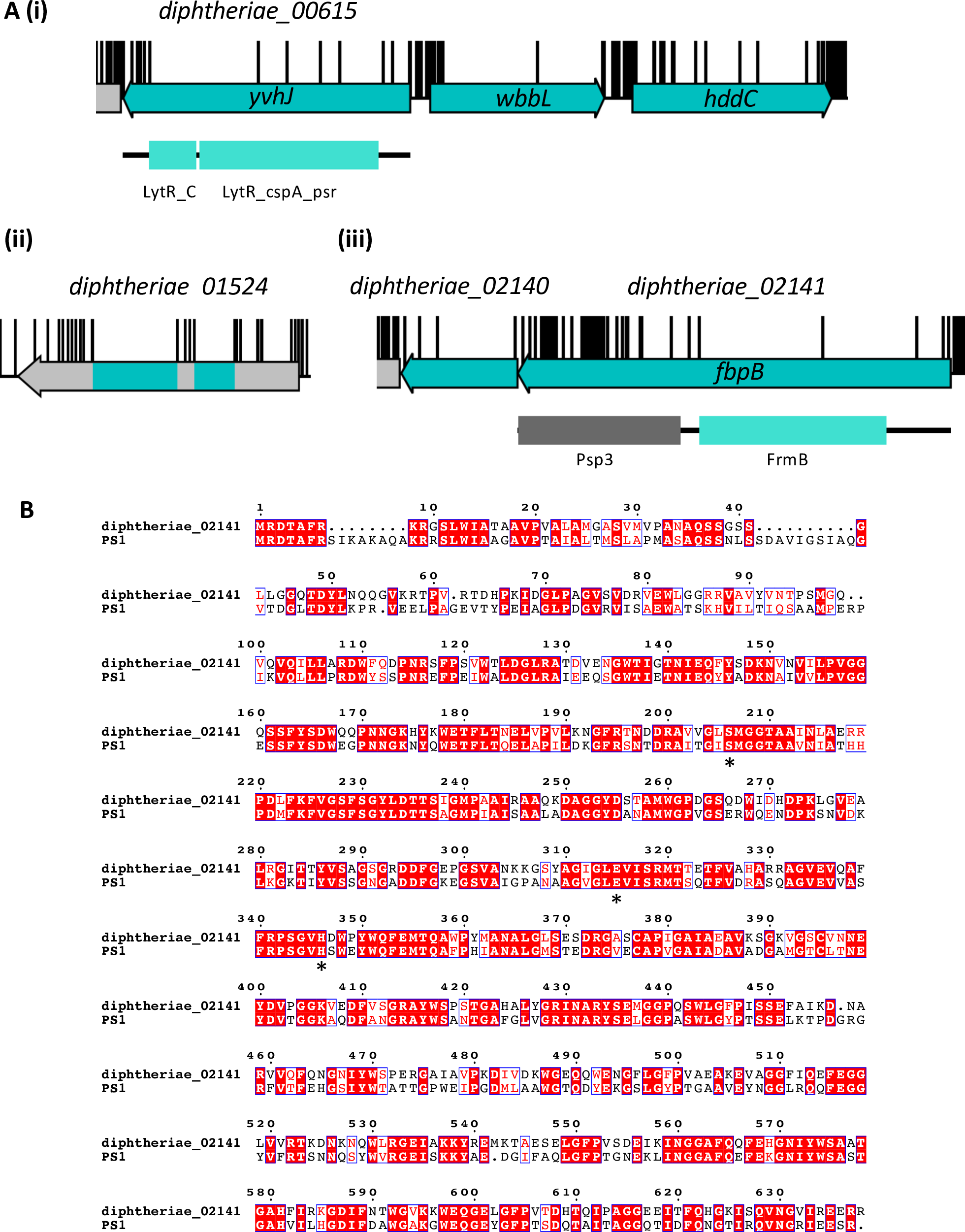

Supplement: S14 Fig — (A) Transposon insertion frequency within the genes of essential proteins detected in the secreted fraction. Essential genes and regions are highlighted in teal. Transposon insertion sites are represented by black bars and capped at a frequency of 1. Protein domains were predicted using the NCBI’s conserved domain (CD-) search [91], and are displayed beneath the gene tract. (B) Sequence alignment of the amino acids of diphtheriae_02141 and PS1 from C. glutamicum (WP_011015455.1), figure generated using ESPript [129]. The conserved catalytic triad are highlighted with (*). (TIF) [file pgen.1010737.s026.tif]

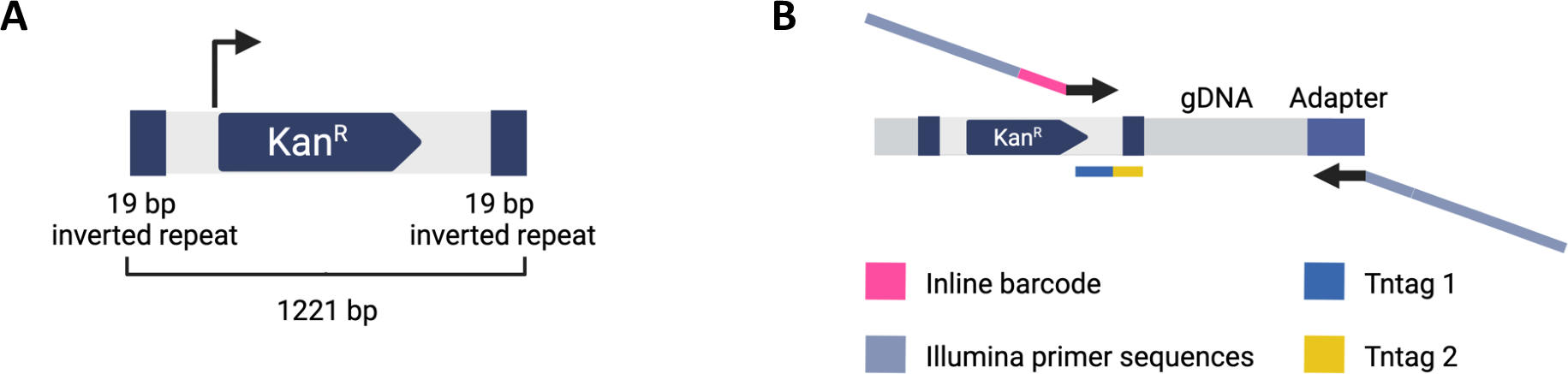

Supplement: S15 Fig — (A) Schematic of the commercially available mini EZ-Tn5 transposon. (B) Schematic of the PCR step that introduces the necessary barcodes and sequences for Illumina sequencing. An inline index barcode is introduced to differentiate between samples and to stagger the start of the transposon sequence during amplicon sequencing. Following successful identification of the inline index barcode during sequence data analysis, the transposon sequence is identified in two steps: Tntag1 (blue) and Tntag2 (amber), which correspond with the primer binding site and the remaining transposon sequence immediately downstream, respectively. Discrepancies between Tntag1 and Tntag2 reveal mis-priming errors during sample preparation for sequencing; these tags correspond with”transposon check 1” and “transposon check 2” described in Table 1. (TIF) [file pgen.1010737.s027.tif]
