# Supplementary material for: A multiomic approach to defining the essential genome of the globally important pathogen Corynebacterium diphtheriae
Source: PLoS Genet. 2023 Apr 26;19(4):e1010737. doi: 10.1371/journal.pgen.1010737 (PMC10166564; doi:10.1371/journal.pgen.1010737)
Supplement: S7 Fig — Heatmap showing the presence/absence of protein orthologs of the 358 essential genes (x-axis) identified in C. diphtheriae in the genomes of 140 representative Corynebacterium species (y-axis) adapted from Dover et al. (2021). Blocks are coloured blue according to percentage identity, above a threshold of 30%, with darker shading corresponding with a higher percentage identity shared with the C. diphtheriae query gene. (PDF) [file pgen.1010737.s019.pdf]

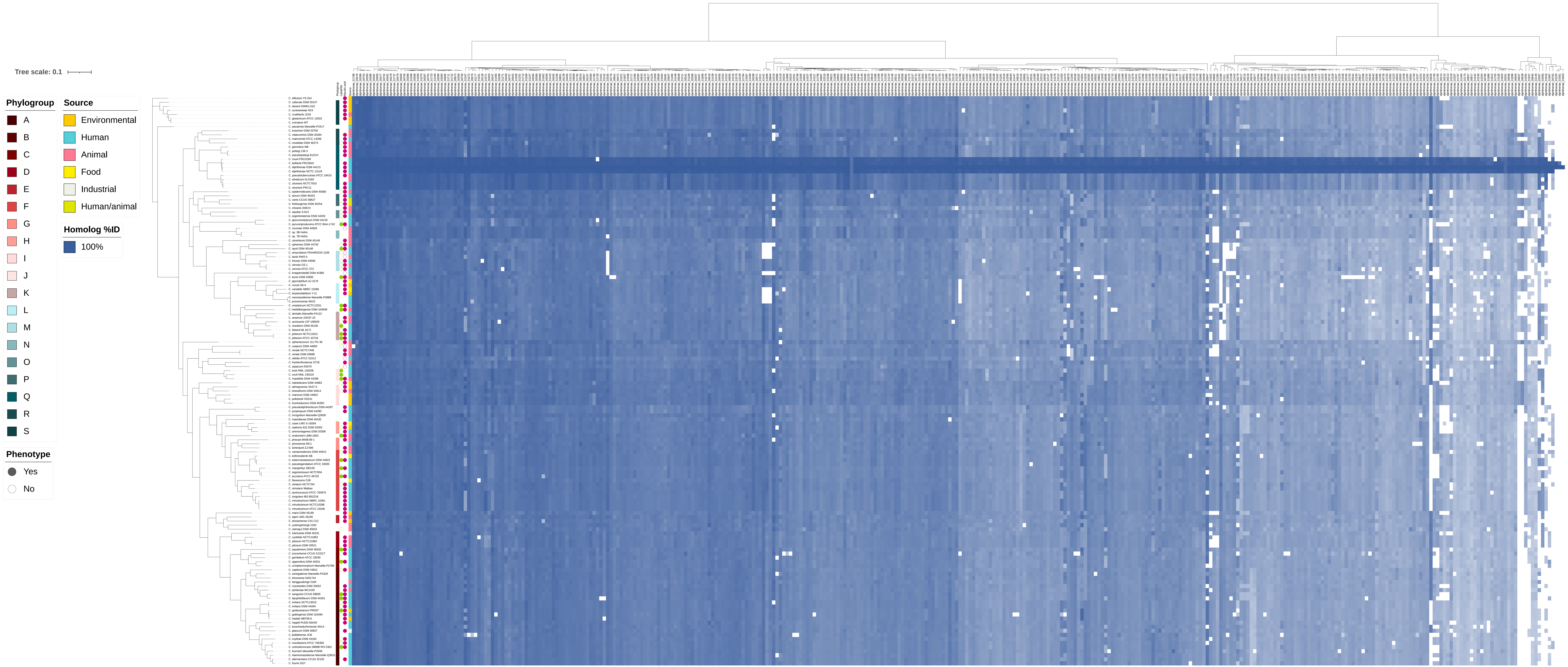
Tree scale: 0.1
Phylogroup
A
B
C
D
E
F
G
H
I
J
K
L
M
N
O
P
Q
R
S
Source
Environmental
Human
Animal
Food
Industrial
Human/animal
Homolog %ID
100%
Phenotype
Yes
No
