# Supplementary material for: A multiomic approach to defining the essential genome of the globally important pathogen Corynebacterium diphtheriae
Source: PLoS Genet. 2023 Apr 26;19(4):e1010737. doi: 10.1371/journal.pgen.1010737 (PMC10166564; doi:10.1371/journal.pgen.1010737)
Supplement: S8 Fig — Homologs were identified by a blastp search and coloured in blue according to the percentage identity to the respective query (C. diphtheriae ISS 3319) gene. Note “dapL” is likely a mis-annotation of “dapC” based on protein alignment analyses. (PDF) [file pgen.1010737.s020.pdf]

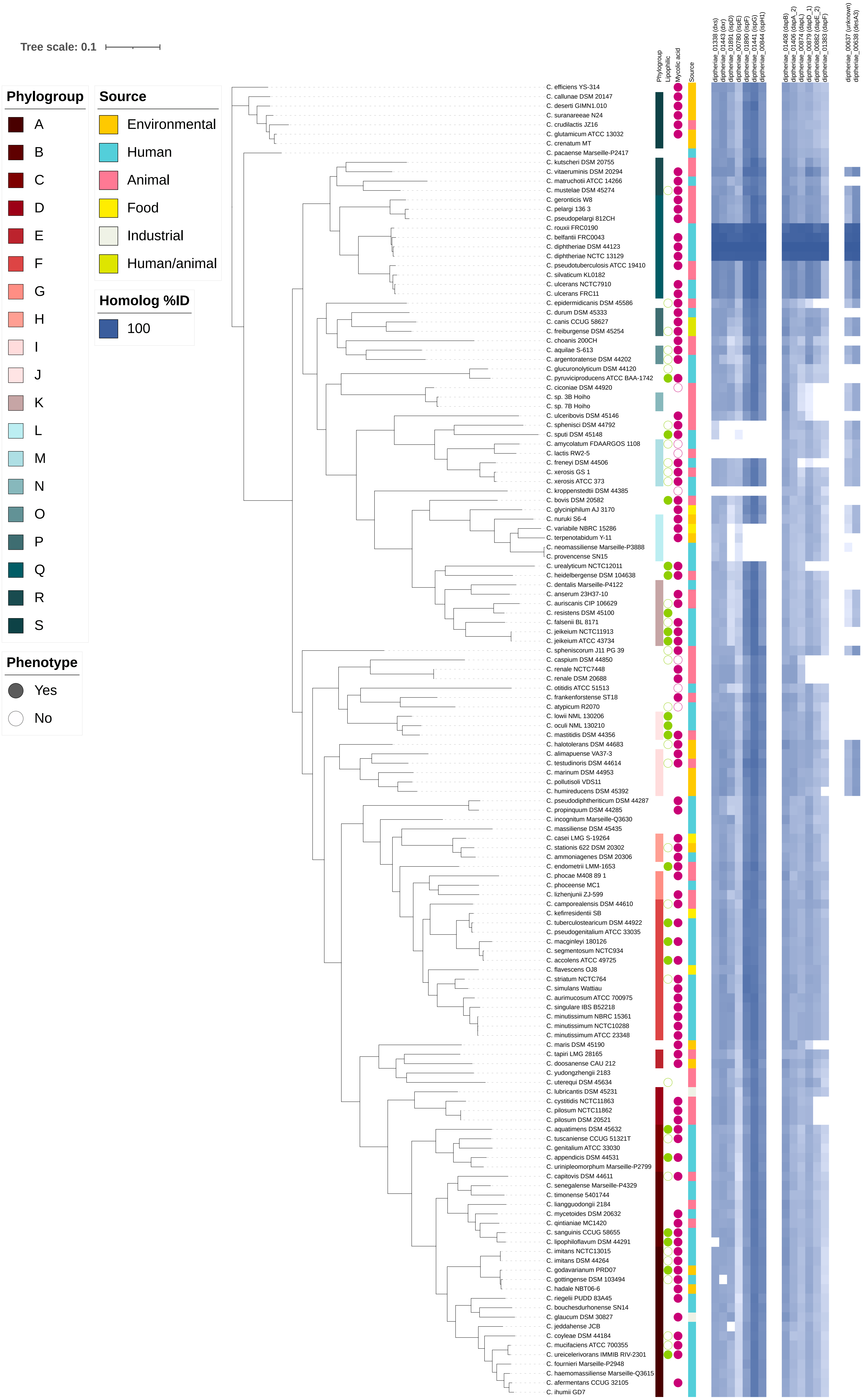

Tree scale: 0.1
Phylogroup
A
B
C
D
E
F
G
H
I
J
K
L
M
N
O
P
Q
R
S
Source
Environmental
Human
Animal
Food
Industrial
Human/animal
Homolog %ID
100
Phenotype
Yes
No
Phylogroup
Lipophilic
Mycolic acid
Source
diptheriae_01338 (dxs)
diptheriae_01443 (dxr)
diptheriae_01891 (ispD)
diptheriae_00780 (ispE)
diptheriae_01890 (ispF)
diptheriae_01441 (ispG)
diptheriae_00844 (ispH1)
diptheriae_01408 (dapB)
diptheriae_01406 (dapA_2)
diptheriae_00874 (dapL)
diptheriae_00879 (dapD_1)
diptheriae_00882 (dapE_2)
diptheriae_01383 (dapF)
diptheriae_00637 (unknown)
diptheriae_00638 (desA3)
C. efficiens YS-314
C. callunae DSM 20147
C. deserti GIMN1.010
C. suranareeae N24
C. crudilactis JZ16
C. glutamicum ATCC 13032
C. crenatum MT
C. pacaense Marseille-P2417
C. kutscheri DSM 20755
C. vitaeruminis DSM 20294
C. matruchotii ATCC 14266
C. mustelae DSM 45274
C. gerontics W8
C. pelargi 136 3
C. pseudopelargi 812CH
C. rouxii FRC0190
C. belfantii FRC0043
C. diphtheriae DSM 44123
C. diphtheriae NCTC 13129
C. pseudotuberculosis ATCC 19410
C. silvaticum KL0182
C. ulcerans NCTC7910
C. ulcerans FRC11
C. epidermidicanis DSM 45586
C. durum DSM 45333
C. canis CCUG 58627
C. freiburgense DSM 45254
C. choanis 200CH
C. aquilae S-613
C. argentoratense DSM 44202
C. glucuronolyticum DSM 44120
C. pyruviciproducens ATCC BAA-1742
C. ciconiae DSM 44920
C. sp. 3B Hoiho
C. sp. 7B Hoiho
C. ulceribovis DSM 45146
C. sphenisci DSM 44792
C. sputi DSM 45148
C. amycolatum FDAARGOS 1108
C. lactis RW2-5
C. freneyi DSM 44506
C. xerosis GS 1
C. xerosis ATCC 373
C. kroppenstedtii DSM 44385
C. bovis DSM 20582
C. glyciniphilum AJ 3170
C. nuruki S6-4
C. variabile NBRC 15286
C. terpenotabidum Y-11
C. neomassiliense Marseille-P3888
C. provencense SN15
C. urealyticum NCTC12011
C. heidelbergense DSM 104638
C. dentalis Marseille-P4122
C. anserum 23H37-10
C. auriscanis CIP 106629
C. resistens DSM 45100
C. falsenii BL 8171
C. jeikeium NCTC11913
C. jeikeium ATCC 43734
C. sphensicorum J11 PG 39
C. caspium DSM 44850
C. renale NCTC7448
C. renale DSM 20688
C. otitidis ATCC 51513
C. frankenforstense ST18
C. atypicum R2070
C. lowii NML 130206
C. oculi NML 130210
C. mastitidis DSM 44356
C. halotolerans DSM 44683
C. alimapuense VA37-3
C. testudinoris DSM 44614
C. marinum DSM 44953
C. pollutisoli VDS11
C. humireducens DSM 45392
C. pseudodiphtheriticum DSM 44287
C. propinquum DSM 44285
C. incognitum Marseille-Q3630
C. massiliense DSM 45435
C. casei LMG S-19264
C. stationis 622 DSM 20302
C. ammoniagenes DSM 20306
C. endometrii LMM-1653
C. phocae M408 89 1
C. phoceense MC1
C. lizhenjunii ZJ-599
C. camporealensis DSM 44610
C. kefirresidentii SB
C. tuberculostearicum DSM 44922
C. pseudogenitalium ATCC 33035
C. macginleyi 180126
C. segmentosum NCTC934
C. accolens ATCC 49725
C. flavescens OJ8
C. striatum NCTC764
C. simulans Wattiau
C. aurimucosum ATCC 700975
C. singulare IBS B52218
C. minutissimum NBRC 15361
C. minutissimum NCTC10288
C. minutissimum ATCC 23348
C. maris DSM 45190
C. tapiri LMG 28165
C. doosanense CAU 212
C. yudongzhengii 2183
C. uterequi DSM 45634
C. lubricantis DSM 45231
C. cystitidis NCTC11863
C. pilosum NCTC11862
C. pilosum DSM 20521
C. aquatimens DSM 45632
C. tuscaniense CCUG 51321T
C. genitalium ATCC 33030
C. appendicis DSM 44531
C. urinipleomorphum Marseille-P2799
C. capitovis DSM 44611
C. senegalense Marseille-P4329
C. timonense 5401744
C. liangguodongii 2184
C. mycetoides DSM 20632
C. qintianiae MC1420
C. sanguinis CCUG 58655
C. lipophiloflavum DSM 44291
C. imitans NCTC13015
C. imitans DSM 44264
C. godavarianum PRD07
C. gottingense DSM 103494
C. hadale NBT06-6
C. riegelii PUDD 83A45
C. bouchesdurhonense SN14
C. glaucum DSM 30827
C. jeddahense JCB
C. coyleae DSM 44184
C. mucifaciens ATCC 700355
C. ureicelerivorans IMMIB RIV-2301
C. fournieri Marseille-P2948
C. haemomassiliense Marseille-Q3615
C. afermentans CCUG 32105
C. ihumii GD7
