# Supplementary material for: A multiomic approach to defining the essential genome of the globally important pathogen Corynebacterium diphtheriae
Source: PLoS Genet. 2023 Apr 26;19(4):e1010737. doi: 10.1371/journal.pgen.1010737 (PMC10166564; doi:10.1371/journal.pgen.1010737)
Supplement: Attachment — Submitted filename: reviewer_responses.docx [file pgen.1010737.s028.docx]

**Comments to the Authors:
Please note here if the review is uploaded as an attachment.**

Reviewer #1: In this article the authors use transposon insertion sequencing, proteomics and data analysis to determine which are the essential genes in Corynebacterium diphtheriae. They also explore the distribution of these genes within the Corynebacterium genus, the presence of the essential proteins in the Diphtheriae vaccine, and they compare the essential genome of C. diphtheriae with those of Mycobacterium tuberculosis and Corynebacterium glutamicum.

The approaches used in the article are appropriate, the results presented are novel, the article is well written, and it is of great interest not only to the community that studies diphtheria, but also to the community that studies different members of Corynebacteriales.

However, I believe that some points should be addressed before publication:

Line 163. How did you choose the threshold of 12? Is it arbitrary? It should be stated.

A threshold of both 4- and 12-fold likelihood for belonging to one mode (essential) vs the other (non-essential) mode have been previously established in the literature as an appropriate threshold for identifying essential genes. We opted for a more stringent and therefore conservative threshold of 12-fold given the lack of biological information for the *C. diphtheriae* ISS 3319 genome.

We have added the citations for literature that use a threshold of 4- or 12-fold change to this sentence of the manuscript and have added the sentence: “We used a conservative and more stringent threshold of 12 to preserve confidence in the list of predicted essential genes”

Line 169. What does it mean to fall in the category “unclear” from a biological point of view? Is it really “unclear” the word that describes this group? I guess that, for example, some of these proteins are not essential but the mutants are more sensitive to the environment. I think this should be discussed.

We have added the following text to the manuscript:

“Besides essential domains within a CDS, there are a number of additional explanations for lowering the IIS of a gene (discussed extensively elsewhere [refs]), such as (1) if a gene, when disrupted, has a severe fitness cost, such that the mutant is slow-growing or forms a small colony on a plate, resulting in the mutant being in low abundance relative to other non-essential mutants within the total mutant pool. (2) If a gene can only tolerate transposon insertions for one orientation of the transposon, for example as a consequence of polar effects, resulting in half as many possible insertions and lowering the IIS. (3) If physiological conditions limit transposon insertion, such as DNA-binding proteins (discussed later). However, in all these scenarios these genes are non-essential and we did not investigate the “unclear” genes further.”

Line 188. I think that the approach you used to identify essential “regions” is correct. But why not also identify essential “domains”? You could have used the NCBI conserved domains tool on the total proteins to delimit the domains, and then the same approach you used for the identification of the essential proteins, in this case on each domain. This would be useful especially in proteins with more than 2 domains, or when the domains have very different lengths. This is just a suggestion, but I really think it would enrich the article. I believe that this would be an incredible contribution to the Corynebacteria community, in particular, for determining the importance of domains of unknown function.

We thank the reviewer for this suggestion and have added the following to the manuscript:

“Finally, we checked for conserved domains (CD) within the essential proteins using NCBI’s CD-search tool [99]. In total, domains were identified for 347/358 proteins, with 17 proteins predicted to have >1 functional domain while no hits were identified for 11 proteins. Of note, nine domains of unknown function were identified within the essential proteome; two of which are predicted to be secreted and five are predicted to be TM proteins, further highlighting gaps in our understanding of essential cell-envelope or secreted protein biology (S. Table 8).”

Line 197. I’m not sure if I understood the approach. Should it say “calculating the proportion of insertion-free regions” instead of “calculating the proportion of insertion-free CDS”?

We have made this change and thank the reviewer for the clarity.

Line 261. What are the annotation artifacts that you mention?

These are genes that have been annotated by the in silico gene annotation tool (prokka) because of the presence of a sizeable open-reading frame with a start codon. However, the lack of homologs of these genes in any other *Corynebacterium* sp., combined with observation of SNPs/indels in the bam file of the tradis data of these 6 genes, suggest the annotation of these genes are annotation errors in the reference genome, and the predicted gene essentiality is a false-positive result caused by the failed mapping of any reads due to local sequence mis-match. To be conservative, the 6 genes that this applies to were caveated from our analysis.

Line 290. It would be useful to indicate which are the groups of genes with differential conservation on Supplementary Figure 7.

These are shown in Supplementary Figure 8.

Line 291. What do you think it means that some genes of the isoprenoid biosynthetic pathway are present but others are absent in some species? Are these genes shared with other pathways? Is it possible that you did not identify the genes because of the approach you used or because the genomes are not complete?

The use of reference genomes made up of contigs is certainly one possibility for the failure to identify a homolog in a given genome, and this may well be the explanation for individual events of missing homologs. However, four of the nine genomes that are lacking *isp* genes are closed genomes. Where there are phylogenetic patterns of absent biosynthetic pathways it is possible that an alternate biosynthetic pathway exists, such as the mevalonate pathway, which is an alternate pathway for the synthesis of isoprenoids in some bacteria.

Line 338. In order to check the absences of the essential genes in Mtb you should try more sophisticated approaches than blastp, like Dali searches against the Alphafold database (<http://ekhidna2.biocenter.helsinki.fi/dali/>). I tried it for a couple of the atp proteins you did not identify using blastp and I obtained good results.

This approach has its merits and may indeed find positive hits where blastp might fail, however, Dali also has its limitations, such as identification of a conserved structural fold is not necessarily evidence for a functional analogue. For example, two ATPases may be structurally similar but this does not mean that they are functionally related We have acknowledged the limitations of blastp within this paper with the example of *ftsL*_2, however, structural conservation is beyond the scope of this paper.

Line 336. I think that you should also review the localization of the total of the proteins and compare these results with those of the essential ones. Membrane proteins in Corynebacteriales are not well characterized, so it would be very interesting to know what proportion of them are essential proteins.

We thank the reviewer for their feedback. The predicted localisation of the total proteome has already been published by Sangal *et al.* (DOI: [10.1186/s12864-015-1980-8](https://doi.org/10.1186/s12864-015-1980-8)) and this information is presented in a column of S. Table 8. However, we have added an additional Supplementary Table (S. Table 7) highlighting the predicted localisation of the essential proteins and have included an additional Supplementary Figure 11 that displays the localisation of the essential proteins in more detail.

Line 391. Which are the essential proteins not detected by the proteome? You mention that many are associated by cell envelope but I could not find this information. For the proteins that are not associated with the cell envelope, what are the reasons why you may not have detected them in the proteomics analysis? You only mention silencing by DNA-binding proteins, but you should mention the limitations of the approach considering that you did not detect a large fraction of the essential proteins.

The proteomics approach was able to detect 286 essential proteins out of 356. Only 70 were not detected by mass spectrometry. 36 proteins are located to the bacterial cell surface and 27 are cytoplasmic proteins, 5 secreted proteins and 2 with ambiguous localization.

Some low abundant proteins might be below the detection limit of the instrument. This can be significantly influenced by many parameters such as chromatographic conditions, complexity of the samples or the separation efficiency of the column.

Additionally, proteins form the bacterial cell envelope are often subjected to various post-translational modifications, such as glycosylation, and phosphorylation which makes them barely accessible for trypsin. Those PTMs can also affect the ionization efficiency of resulting peptides. Both aspects make them difficult to detect by mass spectrometry-based methods.

Proteins which are not located in the cell envelope, such as cytoplasmic proteins, can also have PTMs and therefore underlie the same conditions as mentioned above.

The following sentence has been added to the manuscript: “Similarly, proteins of low abundance, or with post-translational modifications that impede trypsin digestion, may also be missed by our analysis.” We thank the reviewer for their feedback.

Line 407. In Table 3, why did you present the abundance of the essential proteins as a fraction of the total? I am not sure it is the best format. I consider it is very important to show that more than half of the essential secreted proteins are uncharacterized, and this information is diluted when you present it in that format.

The data have been presented as both a table and a figure (Fig. 5B) demonstrating the protein abundance, essentiality, and functional categorization for clarity to the reader and to highlight this point.

Line 447. In order to provide some context, I think you should mention what is the total amount of proteins present in the diphtheria vaccines you are referring to.

We have added the following sentence: “The vaccine contained a total of 130-435 proteins depending upon the country of origin, with 65 proteins shared by all vaccines.”

Line 537. You mention that the plasmid was an artifact. Can you explain how you determined that?

We tried multiple ways to isolate the plasmid, and to detect its presence within the strain, but failed to do so. Following an investigation into the origins of the plasmid, we learned that the sequence arose from a contaminant during sequencing by microbesNG: we discovered that this plasmid was present in another species that was being sequenced by the service at the time. We are in the process of trying to remove the plasmid sequence from the NCBI database.

Line 579. .gbk files are not provided anymore. I guess you meant .gff files.

We apologise for contradicting the reviewer, but the genbank files were downloaded as described, and protein sequences extracted using the tools as described.

Line 633. I would appreciate it if you could comment on the differences you found in the comparison between strains ISS3319 and NCTC 13129. I guess most of them are associated with the synthesis of the toxin, but I’m curious about it.

The vast majority of coding sequences were highly similar but we acknowledge there are differences that may present caveats for the mass spectrometry analysis and have added the following to the manuscript: “2076/2257 (92%) annotated *C. diphtheriae* ISS 3319 genes had a homolog in *C. diphtheriae* NCTC 13129. The vast majority possessed ≥90% identity (1948/2076) and/or ≥90% coverage (1944/2076), therefore we deemed the NCTC 13129 proteome database suitable for analysis, with caveats discussed below.”

Essential proteins that were overlooked in the analysis (*diphtheriae_01603* and *diphtheriae_02320*) due to the methodology had already been highlighted in the text. We did not pursue genomic differences further as this was beyond the scope of this manuscript and has in part been reported in the literature previously (Sangal *et al.* (DOI: [10.1186/s12864-015-1980-8](https://doi.org/10.1186/s12864-015-1980-8)) S. Table 3). However, we did observe in some preliminary analysis, not included as part of the manuscript, that COG classification of genes exclusive to each strain were predominantly COG groups L, S, X and V, corresponding with “Replication, recombination and repair”, “function unknown”, “mobilome: prophages, transposons” and “defence mechanisms”; which is consistent with reports of high horizontal gene transfer for the genus.

Line 652. Did you check if the genes identified as orthologous can actually be concatenated? Did you do any topology test before concatenation?

We apologise for the confusion here, for clarity, concatenated in this context is referring to the sequential appending of protein FASTA data into a single multi-sequence-alignment file. The concatenated output still maintains individual protein partitioning, i.e. no protein sequences were artificially fused into a single coding sequence.

Line 658. In the data availability statement you indicate that the annotated genome can be accessed at the ENA site, but I could not find this information. It is extremely important that you provide the annotations of the genome so the community can make use of the data you generated in this article. It is also of huge importance that every time that you mention a gene like “diphtheriae_XXXXX” you also include the corresponding DIP gene tag, otherwise it is very hard to know which is the gene you are referring to.

We thank the reviewer for identifying this as this was an oversight on our part. The annotation file has been made available through Figshare ([10.6084/m9.figshare.22332187](https://doi.org/10.6084/m9.figshare.22332187)) and we have made the following edit to the manuscript:.

“Transposon sequencing data are available for download at the European Nucleotide Archive (accession: PRJEB56349). The genome annotation used in this analysis is available for download here: [10.6084/m9.figshare.22332187](https://doi.org/10.6084/m9.figshare.22332187).”

We also thank the reviewer for their suggestion of DIP numbers and have added DIP numbers throughout at the first instance of each diphtheriae_XXXX number.

Supplementary Figures 6 and 12. Which are the genes indicated with numbers? These numbers should be specified in the legend or not provided at all.

The numbers are an output generated by the WebFlags tool. There are too many to be provided in the figure legend and we have provided these numbers in a new supplementary information document and added to the Supplementary Figure legends: “The remaining predicted gene functions associated with each number are provided in Supplementary Information.” We thank the reviewer for highlighting this.

Reviewer #2: Goodall et al. constructed and analyzed a high-density transposon directed insertion library in C. diphteria to identify the essential genome. The authors find 341 essential genes that largely correspond to essential genes of other species within the Actinobacteriota.
This work contains an impressively dense library and a very thorough analysis. I only have a few questions that are mainly related to the EZ-Tn5 Kan-2 kit that is used in the study. I would appreciate a couple of sentences and maybe a small supplementary figure what the effect of an insertion within a coding sequence is (i.e., How large is the insertion? Does it contain outward facing promotors?).

We thank the reviewer for their comments and have provided a schematic of the transposon in supplementary figure 15A with some additional detail.

The EZ-Tn*5* transposon contains a promoter for the expression of the kanamycin-resistance *aph* gene. While transcriptional (and translational) read-out from mini-Tn*5* transposons has been reported previously (Goodall *et al.* 2018, Fig. 4 DOI: <https://doi.org/10.1128/mBio.02096-17> ) we have not verified the transcriptional read out from this transposon within *C. diphtheriae*. However, the presence of polar transposon insertion profiles in a handful of genes would strongly suggest the same is true of this system as documented in *E. coli*.

1. How does the insertion affect operon structures? Is it possible that a non-essential gene is classified as essential because the insertion disrupts transcription of another gene within the operon?

This has been discussed in other papers in the literature but in brief this depends on both the transposon and genomic context at the point of insertion. In this library, the data suggests that expression of downstream essential genes is maintained when an upstream gene of the same operon is disrupted. Examples of this include the *wecA* (*diphtheriae*_00950) and *ispE* (*diphtheriae*_00780) operons.

2. Can you detect essential RNAs?

Yes and no… the tmRNA ssrA is clearly essential, however the rRNA diphtheriae_00691 shows clear insertion orientation bias – a phenomenon that has been observed in many TIS datasets for numerous species but does not yet have a detailed explanation. We chose to exclude analysis of non-coding regions from this manuscript because they can be difficult to classify for 3 reasons (1) they require annotation (2) they are often smaller in size and beyond the resolution detection level and (3) sometimes have curious insertion patterns. Overall, we deemed these beyond the scope of this manuscript.

3. Are there duplicate genes in the genome that are potentially essential but would be missed by this approach since either can the deleted individually?

This raises a really interesting debate. We did not investigate any non-essential genes further, under the assumption that if they can be disrupted they are likely not essential for viability. However, it is possible (and probable) that there will be a pair (or more) of genes that together provide an essential function for the cell but share functional overlap such that a single gene can be deleted. A well-documented example of this is *mrcA*/*mrcB* in *E. coli*, which encode the penicillin binding proteins PBP1A and PBP1B, respectively. Genes like these would certainly be missed in a screen like this, and indeed in any conventional screen to identify essential genes. This opens the debate of whether a gene can be considered essential if there are conditions where it can be deleted. For this reason, our data are caveated with the statement “essential under the conditions tested”, and we did not investigate the genes identified as “non-essential” further.

4. Lines 166-169: Have the 115 genes that are classified as unclear been analyzed further? I agree that the insertion density within the resA_2 gene (Fig 1) seems lower than for the others but based on the insertion pattern resA_2 doesn’t look like an essential gene to me. Also, the gene name indicates that there are more copies of this gene on the genome. Could that affect the results?
The unclear genes were not analyzed further, there are a number of reasons for a gene to have an ambiguous insertion index score and text to discuss this has been added to the manuscript in some more detail:

“Besides essential domains within a CDS, there are a number of additional explanations for lowering the IIS of a gene (discussed extensively elsewhere [refs]), such as (1) if a gene, when disrupted, has a severe fitness cost, such that the mutant is slow-growing or forms a small colony on a plate, resulting in the mutant being in low abundance relative to other non-essential mutants within the total mutant pool. (2) If a gene can only tolerate transposon insertions for one orientation of the transposon, for example as a consequence of polar effects, resulting in half as many possible insertions and lowering the IIS. (3) If physiological conditions limit transposon insertion, such as DNA-binding proteins (discussed later). However, in all these scenarios these genes are non-essential and we did not investigate the “unclear” genes further.”

There were a handful of genes with (>1) number in addition to the gene name. The effect of functional duplication (and implications for essential gene analysis) is addressed above, however, the presence of a number in the gene name is not necessarily an indication of functional redundancy (for example *ftsL_1* and *ftsL_2,* which were discussed in the manuscript).

5. Is there a sequence bias for insertion? Could some of the ‘unclear’ genes be explained due to this bias?

Tn*5* insertion is not limited to specific motifs unlike the himar1 mariner transposon which requires an AT dinucleotide. There are some reports of insertion preference being weakly influenced by %GC or localised DNA shape, but the Tn*5* transposon, and derivatives, are considered to have insufficient bias to affect essential gene classification. We have added the following sentence to the manuscript and thank the reviewer for their comment.

“Using previously described methods [56,62], we constructed a transposon mutant library in *C. diphtheriae* strain ISS 3319 using a mini-Tn*5* transposon as they are considered to have negligible insertion bias for the analysis of essential genes [63]”

6. Lines 196-201: Does this analysis for essential domains only work for the N-terminus of a gene or also for the C-terminus?

This depends entirely on your transposon, the ability to maintain downstream protein translation, and whether a N-terminally truncated essential protein is still functional. However, an example of this has been demonstrated in *grpE* of *E. coli*, and for this dataset we observed a similar insertion pattern within *diphtheriae*_02047.

Interestingly, analysis done in *Mycobacterium* *tuberculosis* by Zhang *et al* (DOI: <https://doi.org/10.1371/journal.ppat.1002946>) demonstrated C-terminal essential regions to be just as prevalent as N-terminal essential regions.

7. Lines 202-253: Would the insertion of a transposon in the middle of the gene split the protein into two independent proteins? A MurJ-like protein and a Pseudokinase protein.

Theoretically yes, if the transposon is able to re-initiate translational read-out from the transposon, or if the upstream endogenous promoter initiates transcription that is maintained across the full length of the transposon and downstream gDNA, both of these scenarios could result in protein expression of the downstream peptide and is certainly a possibility for the MviN pseudokinase domain. An example of this (*grpE* in *E. coli*) has been described in the literature previously (<https://doi.org/10.1128/mBio.02096-17>).

It is beyond the scope of this manuscript, but one of the things we have considered is whether MviN might be functional and expressed *in vivo* from >1 start codon, in a similar fashion to ArcB in *E. coli* (DOI: [10.1016/j.molcel.2019.02.017](https://doi.org/10.1016%2Fj.molcel.2019.02.017)), as this hypothesis would fit with the *mviN* transposon insertion profile. ArcB has >1 start codon; both the full length protein CDS and a single domain of the protein (a functional phosphotransfer domain) can be independently expressed from the same CDS. The proposed function of this dual protein expression is to enable regulation of the ArcB phosphotransduction system, or cross-talk with other signal transduction systems. Verification of this would be a substantial piece of work beyond the scope of this manuscript.

8. Fig 1C: The figure looks like transposons are inserted right up to the start codon of nadE. Does the Tn5 transposon contain a transcriptional start and ribosome binding site?

The transposon has a promoter and RBS for expression of the antibiotic resistance cassette, enabling selection of successful transformants. The Tn*5* system has been used widely in transposon mutagenesis screens in many, diverse, bacterial species so it is well established in the literature.

Please note that the scale of these figures means that bp resolution insertion data for a gene of 834 bp has to be condensed down to ~2-3cm of a page. However, yes it is entirely feasible for a transposon to viably insert immediately upstream of a start codon (and this does occur). The viability of this mutation can be attributed to (1) the transcriptional read-out of the antibiotic selection cassette, (2) the presence of additional putative RBS sequences within the Mini-Tn*5* transposon and (3) the insertion of a mini-Tn*5* transposon system into DNA results in a 9-bp duplication at the site of insertion. This last detail means it’s possible to insert a transposon within 9 bp of the first nucleotide of a start codon while still maintaining that same start codon downstream of the transposon. For review, a really nice early paper on high-throughput transposon mutagenesis screening even showed this level of resolution can be used to identify start codons (Christen *et al.* 2011; Fig 2H: <https://pubmed.ncbi.nlm.nih.gov/21878915/> ).

The insertion data will be made publicly available here: <https://tradis-vault.qfab.org/> , for browsing of individual insertions if of interest.

9. How are occasional insertions within essential genes explained (i.e., mrpA in Fig S10)?

This has been documented previously in the literature. However as with any biological high-throughput screens, despite assaying something you would expect to be clear-cut like life/death, the output is never perfectly binary. We expect that mutations will occur, such as duplication(s) of regions of the genome, or secondary mutations, both of which will maintain the viability of an otherwise lethal mutation. In both of these scenarios it might become possible to viably disrupt an essential gene with a transposon. However, we expect that secondary, compensatory mutations occur at a sufficiently low frequency to still be able to distinguish “essential” from “non-essential” genes. Additional explanations for occasional insertions include (1) if the genetic material of dead cells/failed mutants was pooled when colonies were scraped from the plate during construction of the library, albeit at a very low abundance. (2) In the example of *grpE* in *E. coli* (Goodall *et al.* DOI: <https://doi.org/10.1128/mBio.02096-17>), whereby transposon insertion has occurred at a structurally insignificant position of the essential protein, and the essential protein can still be expressed as 2 peptides. (3) If the gene is not-essential and is a false positive result, but transposon insertion has been occluded by DNA-binding proteins resulting in very sparse transposition. (4) If the gene is technically non-essential (it can viably be deleted), but loss of the gene has a severe fitness cost, such that either (a) the resulting mutant has a very small colony phenotype or (b) a very slow growth phenotype, such that is in lower abundance within the overall pool of mutants, for example *fabH* mutants of *E. coli*.

In short, despite the expected outcome of being able to simply classify essential from non-essential genes, biology can be beautifully grey and a challenge to mathematically classify.

Reviewer #3: This paper from Goodall and co-workers describes the use of transposon insertion sequencing (TnSeq/TraDIS) to characterize the essential genome of the pathogen Corynebacterium diptheriae (Cdip). This bacterium belongs to the Corynebacteriales order that includes other pathogens like Mycobacterium tuberculosis (Mtb) and model organisms like Mycobacterium smegmatis and Corynebacterium glutamicum (Cglu). These bacteria have a unique cell envelope consisting of multiple layers including the peptidoglycan (PG), arabinogalactan (AG), and mycomembrane (MM). Unlike most model bacteria, they also grow via tip extension. Thus, understanding the biology of these bacteria is important for addressing fundamental questions about cell growth as well as for practical reasons of therapeutic development.

The main advance of this paper is the generation of the first high-density transposon library in a pathogenic Corynebacterium and using it to determine its essential genome and compare it to other results from Mtb and Cglu. The paper is very well written and the bioinformatics is well done. The dataset generated will be highly valuable to the field.

Major critique:
Where the paper fall a little short in my opinion is the lack of genetic validation of any of the inferences drawn from the TnSeq data. The results would be much stronger if some of the observations were followed up with genetic experiments. For example, the authors claim that pknB is non-essential in Cdip unlike Mtb and that the mrp operon is essential and a unique transport pathway distinct from Mtb. My understanding is that tools are available to engineer Cdip, so adding some genetic validation to strengthen the results should be feasible.

We thank the reviewer for their kind feedback and have constructed a *C. diphtheriae* ISS 3319 mutant in *pknB* in support of the TIS data. We have added a supplementary figure indicating that we can independently disrupt *pknB* using alternative methodology in *C. diphtheriae* validating the TIS data; see S. Fig. 5C-D.

And added the following to the manuscript: “In support of the TIS data, we constructed an isogenic *pknB* mutant and confirmed its growth and genotype (S. Fig. 5C-D).”

With the following text added to the materials and methods:

“**Construction of the ISS3319::pK18mobDIP0053 (*pknB* mutant) strain**

Standard techniques for plasmid isolation, transformation and cloning were used throughout [103]. Chromosomal disruption of the *C. diphtheriae* *diphtheriae_00047* (DIP0053) gene was achieved through amplification of a 549 bp internal DNA fragment of *diphtheriae_00047* using PCR from chromosomal template DNA of *C. diphtheriae* ISS 3319 using the following primers: DIP0053Mut_F 5'-aggttgcggtaaaaatgctg-3 '; DIP0053Mut_R 5'-caccctcgaaaggtggttta-3'. The PCR fragment was cloned in to pGEM-T-Easy (Promega) according to the manufacturer’s instructions. The fragment was then excised from pGEM-T-Easy using *Eco*RI and sub-cloned in to dephosphorylated pK18mob cut with *Eco*RI to yield pK18mobDIP0053 [104]. This was then passaged through the non-methylating *E. coli* GM2929. One microgram of unmethylated plasmid isolated from *E. coli* GM2929 was used to transform *C. diphtheriae* using GenePulser II (Bio-Rad). Electroporated cells were allowed to recover in 1 ml of Brain Heart Infusion (BHI) broth containing 1% glucose for 2 h at 37°C. The recovered cells were plated on to BHI medium containing kanamycin (50 µg/ml) and incubated for 18 hours at 37°C. The plasmid pK18mob cannot replicate in *C. diphtheriae*, such that kanamycin-resistant *C. diphtheriae* are carrying the vector integrated via recombination in to the chromosomal *diphtheriae_00047* (DIP0053) locus. The stable integration was checked by PCR using primers internal to pK18mob (pK18mob_CheckF: 5’-taggtgttagcggaccatcc-3’) and a primer internal to the *diphtheriae_00047* (DIP0053) CDS (DIP0053KO_CheckR: 5’-gttttcccagtcacgacgtt-3’) using genomic DNA as a template, which yields a 327bp fragment in the mutant strain, that is absent in the WT strain (S. Fig. 5C).’

Minor points:
1) The tables require more detail in the legends. It is not clear what “inline barcode match”, “transposon check 1 or 2” refer to in Table 1. Insertions density should be defined in Table 2 legend.
We apologise for the oversight and thank the reviewer for highlighting this. More details have been added to the manuscript as follows:

The following footnote has been added to Table 1: ^a^ These barcodes/tags correspond with the sequential pattern matching and trimming positions of the raw sequencing data during data processing. Please refer to S. Fig. 15B for further detail.

The following footnote has been added to Table 2: ^b^ Insertion density is the genome size divided by the number of insertions or mutants reported for that genome to give an approximation of 1 insertion every X bp as a measure of overall library density.

**A**

**B**

**Supplementary Figure 15.** **Details of the transposon and transposon library sample preparation for sequencing.** (A) Schematic of the commercially available mini EZ-Tn*5* transposon used in this study. (B) Schematic of the PCR step that introduces the necessary barcodes and sequences for Illumina sequencing. An inline barcode is introduced to differentiate between samples and to stagger the start of the transposon sequence during amplicon sequencing. Following successful identification of the inline barcode during sequence data analysis, the transposon sequence is identified in two steps: Tntag1 (blue) and Tntag2 (amber), which correspond with the primer binding site and the remaining transposon sequence immediately downstream, respectively. Discrepancies between Tntag1 and Tntag2 reveal mis-priming errors during sample preparation for sequencing; these tags correspond with ”transposon check 1” and “transposon check 2” described in Table 1.

2) For the predictions of secreted proteins, it would be nice to add Tat transport and sortase signals.

We thank the reviewer for their feedback, we have added the following sentence “the specific predicted secretion pathways are shown in S. Fig. 11” to the manuscript and an additional supplementary figure:

**Total Essential Proteome**

**Transmembrane**

**Secreted**

**Supplementary Figure 11. Predicted localization of essential proteins**

The predicted localization of essential proteins adapted from Sangal *et al.* (2015) shown for the total proteome (top), the predicted transmembrane proteins (left, 51) and predicted secreted proteins (right, 25). Abbreviations: Cyt, cytoplasmic; TM, transmembrane; Sec, secreted; Lipo, lipoprotein; NC, non-classical secreted protein; Spi, SpI type signal peptide; Tat, Tat signal peptide; Amb, ambiguous.
